# Prion Protein Interaction with Soil Humic Substances: Environmental Implications

**DOI:** 10.1371/journal.pone.0100016

**Published:** 2014-06-17

**Authors:** Gabriele Giachin, Joanna Narkiewicz, Denis Scaini, Ai Tran Ngoc, Alja Margon, Paolo Sequi, Liviana Leita, Giuseppe Legname

**Affiliations:** 1 Laboratory of Prion Biology, Department of Neuroscience, Scuola Internazionale Superiore di Studi Avanzati (SISSA), Trieste, Italy; 2 Life Science Department, University of Trieste, Trieste, Italy; 3 Consiglio per la Ricerca e Sperimentazione in Agricoltura (CRA), Gorizia, Italy; USGS National Wildlife Health Center, United States of America

## Abstract

Transmissible spongiform encephalopathies (TSE) are fatal neurodegenerative disorders caused by prions. Animal TSE include scrapie in sheep and goats, and chronic wasting disease (CWD) in cervids. Effective management of scrapie in many parts of the world, and of CWD in North American deer population is complicated by the persistence of prions in the environment. After shedding from diseased animals, prions persist in soil, withstanding biotic and abiotic degradation. As soil is a complex, multi-component system of both mineral and organic components, it is important to understand which soil compounds may interact with prions and thus contribute to disease transmission. Several studies have investigated the role of different soil minerals in prion adsorption and infectivity; we focused our attention on the interaction of soil organic components, the humic substances (HS), with recombinant prion protein (recPrP) material. We evaluated the kinetics of recPrP adsorption, providing a structural and biochemical characterization of chemical adducts using different experimental approaches. Here we show that HS act as potent anti-prion agents in prion infected neuronal cells and in the amyloid seeding assays: HS adsorb both recPrP and prions, thus sequestering them from the prion replication process. We interpreted our findings as highly relevant from an environmental point of view, as the adsorption of prions in HS may affect their availability and consequently hinder the environmental transmission of prion diseases in ruminants.

## Introduction

Prions are proteinaceous infectious agents causing a heterogeneous group of invariably fatal neurodegenerative disorders denoted as transmissible spongiform encephalopathies (TSE) or prion diseases. Creutzfeldt-Jakob disease (CJD) is the most common form of TSE in humans whereas animal TSE include scrapie in sheep and goat, chronic wasting disease (CWD) in cervids and bovine spongiform encephalopathy (BSE) in cattle [1]. The central event leading to prion formation is the conformational conversion of the ubiquitously expressed cellular form of the prion protein (PrP^C^) to a misfolded isoform denoted as prion or PrP^Sc^ [2]. Unlike PrP^C^, PrP^Sc^ is amyloidogenic, unusually resistant to proteolytic enzymes and enriched in β-sheet secondary structure motifs [3].

Prion diseases uniquely manifest as sporadic, inherited or iatrogenic, *i.e.* prions are acquired through infectious routes. In ruminants, scrapie and CWD can be transmitted *via* environmental routes, while BSE is transmitted almost exclusively through foodborne carriages [4]. In natural environments, prions are most likely acquired *via* oral intake [5]–[7]; amplification of PrP^Sc^ follows in the lymphoid tissues associated with the gut of the host [8]. In BSE, PrP^Sc^ accumulation has been largely found within the CNS [9] whereas scrapie and CWD have exhibited a widespread prion distribution in different tissues [10], [11]. The PrP^Sc^ tropism observed in sheep and cervids accounts for the facile TSE transmission among these animals, which may disseminate prions *via* multiple excretion routes [12]. The occurrence of endemic scrapie and CWD reported in affected areas points to the presence of environmental reservoirs. It is accepted that soil may harbor prion infectivity –PrP^Sc^ is resistant to biotic and abiotic degradation and can persist in soil for years [13]–[15]. Soil-bound prions retain infectivity, as experimentally validated in intracerebral [16]–[19], oral and intranasal infection studies [20], [21]. PrP^Sc^ bound to soil particle surfaces is mediated by electrostatics and hydrophobic interactions [16], [22], [23]. Prions may interact with other soil constituents such as organic matter (OM). In particular, PrP^Sc^ can interact with humic substances (HS) –currently defined as supramolecular, meta-stable structures of self-assembled molecules held together by multiple weak interactions [24]–[26]. Humic and fulvic acids (HA and FA, respectively) constitute HS. Differently from HA, FA have lower molecular weight, higher functional group density and higher acidity [27]. HS can interact with xenobiotics and proteins and polymerize, forming large molecular ensembles. The interaction between HS and proteins under different experimental conditions has been described as encapsulation of the polypeptides into the inner HS structure [28]–[31]. Previous interaction studies between recombinant prion proteins (recPrP) and HS have shown that HS impact recPrP adsorption, forming insoluble complexes with the protein [32]–[35]. A comparison of epidemiological CWD data with soil clay content in a delimited geographic region (Northern Colorado, Colorado State, USA) has shown that the odds of prion infection in deer are increased in soil areas with high clay content but poor in OM [36]. The interaction of prions with soil OM may be highly environmentally relevant for soils rich in HS, but the mechanism of HS-PrP^Sc^ interaction and the influence of HS on prion infectivity are not well understood.

To provide more clues on the role of HS in limiting prion infectivity, we investigated the adsorption of murine full-length recPrP (MoPrP) to natural HS. We characterized these supramolecular complexes by circular dichroism (CD), optical and atomic force microscopy (AFM). We analyzed how HS adsorbing MoPrP hinder proteolytic degradation, and we evaluated whether HS limit prion conversion by using both a kinetic assay for PrP^Sc^ conversion [37] and a neuronal cell culture model (ScGT1) chronically infected with the Rocky Mountain Laboratory (RML) prion strain [38]. Our findings on HS interacting with MoPrP add evidence for their role as anti-prion complexants. Our study may contribute to better understand the large complexity of environmental factors that impact prion persistence and infectivity in soil.

## Materials and Methods

### Extraction and Purification of Humic Substances

A composite sample of a meadow soil from northeastern Italy was collected at 5–30 cm depth. No specific permissions were required for these sampling activities in this location. The field studies did not involve endangered or protected species. The GPS coordinates of the private land where the soil was collected are 45.9344° in latitude and 13.6136° in longitude. Contact the corresponding authors to request authorization for further use of this material. The soil was a sandy loam Mollic Hapludalf (USDA) composed of 67% sand, 21% silt, 12% clay, 2.2% organic C, 0.21% total N and 10.5 organic C/total N ratio at pH 6.8. Soil HS were extracted according to the International Humic Substances Society (IHSS) guidelines. HA and FA were purified according to [39]. HS quantification was based on the organic carbon content and expressed as µg/mL of HS. The stock solutions of HA and FA were autoclaved and diluted to 0.5 mg/mL in acetate buffer (20 mM NaOAc, 0.005% NaN_3_, pH 5.5). The pH was monitored prior to use.

### MoPrP Expression and Purification

The plasmid pET-11a (Novagen) encoding for the full-length MoPrP(23–230) was kindly provided by Prof. J.R. Requena (University of Santiago de Compostela, Santiago de Compostela, Spain). An overnight culture of *E. coli* Rosetta 2 (DE3) (Novagen) freshly transformed with the plasmid was added at 37°C to 2 L of Zym-5052 medium [40]. Cells were grown in a 2 L fermenter system (Sartorius), harvested after 24 hours and lysed by homogenizer (Panda plus, GEA Niro Soavi). Inclusion bodies were washed and solubilized according to [41]. MoPrP was purified using its octapeptide repeat sequence as natural affinity tag for nickel. MoPrP was loaded onto a 5-mL HisTrap column (GE Healthcare) equilibrated in binding buffer (2 M GndHCl, 500 mM NaCl, 20 mM Tris, pH 8) and eluted with 500 mM imidazole. Subsequently, the protein was purified by reverse phase (Jupiter C4, Phenomenex) and separated using a gradient of 0–95% acetonitrile and 0.1% trifluoroacetic acid. The purified protein was lyophilized and refolded in acetate buffer (25 mM NaOAc, 0.005% NaN_3_, pH 5.5) according to [41].

### Circular Dichroism and SDS-PAGE Gel Experiments

Samples for SDS-PAGE gel experiments consisted of 125 µg/mL MoPrP incubated for 12 hours at 22°C in the presence of increasing amounts of HS (1, 5, 7.5, 10 and 20 µg/mL of HA and FA) in a volume of 50 µL in buffer acetate (25 mM NaOAc, 0.005% NaN_3_, pH 5.5). After incubation, samples were centrifuged at 16,000 g for 45 min to separate the soluble protein from the precipitated fractions. Supernatants and pellets were boiled for 10 min in 4X loading buffer (5 M Urea, 10% SDS, 0.25 M Tris HCl, 0.4 M DTT, 30% Glycerol, 0.04% bromophenol blue, pH 6.8), loaded onto 12% SDS-PAGE gel. Circular dichroism (CD) spectra were recorded over the 195 to 260 nm range using a CD spectrometer (Jasco Inc.) at 22°C. Samples were prepared with 125 µg/mL of MoPrP incubated either in the presence or absence of HS (1, 5, 7.5, 10 and 20 µg/mL of HA and FA) for 6 and 240 hours at 22°C in buffer acetate. After incubation, samples were centrifuged at 16,000 g for 45 min to separate the soluble protein from the precipitate. CD spectra on supernatants were accumulated three times and acquired in triplicate using a 1-mm path-length cell. To estimate the concentration of MoPrP in solution after HS addition, we acquired CD spectra of MoPrP standards (125, 100, 75 and 50 µg/mL) and derived the equations resulting from the linear fitting of the mdeg value at 208 nm *versus* the MoPrP standards. The CD spectra of the samples treated with HA or FA were then normalized in molar ellipticity (θ). Due to the CD intrinsic sensitivity limit, only spectra with a minimum of about −4 mdeg at 208 nm were normalized. CD data were processed by Spectra Manager 1.52.01 and Origin 8.6 software.

### AFM and Optical Microscopy of MoPrP-HS Complexes

Two MoPrP concentrations (25 and 60 µg/mL) were incubated with 5 and 20 µg/mL of HA or FA in 50 µL volume at 22°C for 6 hours. All the samples were prepared by drop casting on a surface of freshly cleaved muscovite mica and left to adhere till total solvent evaporation. All AFM measurements were performed on a standard MFP-3D stand alone Asylum Research AFM (Oxford Instruments, UK) in dynamic mode using commercially available silicon probes (NSG30 cantilever characterized by a spring constant of about 40 nN/nm, resonance frequency of about 340 kHz and tip radius less than 6 nm). Images were acquired in air at a resolution of 512×512 pixels at 1 Hz scan speed. Optical microscopy characterization was performed on a Nikon Eclipse Ti-U inverted microscope. Phase images were recorded with a Nikon Digital Sight DS-2 Mv camera at 1600×1200 pixels resolution on a 20× objective.

### Protease-K Digestion on HS-adsorbed MoPrP

Samples for the digestions consisted of 125 µg/mL of MoPrP incubated in the presence of 20 µg/mL HS at 22°C for 6 hours in a final volume of 50 µL, and then centrifuged at 16,000 g for 45 min to separate the precipitated protein. Pellets were resuspended twice in 50 µL of acetate buffer. Samples were digested with increasing concentrations of Protease-K (PK) (Roche) (1, 5, 10, 25, 50 and 100 µg/mL) for 1 hour at 37°C. The reactions were stopped adding 5 mM of phenylmethylsulfonyl fluoride (PMSF), boiled for 15 min in 4X loading buffer, loaded onto a 12% SPS-PAGE gel and blotted on Immobilion PVDF (Millipore) membrane. Membrane was probed with anti-PrP monoclonal antibody W226 and developed by enhanced chemiluminescence (GE Healthcare). Band intensity was acquired using the UVI Soft software (UVITEC, Cambridge). As non-PK control, we loaded 0.1 µg of MoPrP to avoid signal saturation.

### Monitoring the Kinetics of *in vitro* Fibril Formation by Amyloid Seeding Assay

To monitor the formation of ThT-positive fibrils, we used 50 µg/mL of α-helix folded MoPrP in acetate buffer. To induce protein polymerization we added to the reaction a preformed PrP^Sc^ seed chemically purified from ScGT1 cells [42]. First, the amyloid seeding assay (ASA) was performed incubating the PrP^Sc^ seed with MoPrP for 6 hours, and then HS were added to the reaction. In a second ASA, the seed was incubated with 1 µg/mL HS for 6 hours and then we added MoPrP. MoPrP and MoPrP incubated with 1 µg/mL HS were used as negative controls. ASA was performed in a final volume of 100 µL using a 96-well plate (PerkinElmer). Experiments were performed at least in quadruplicate and the plate was incubated at 37°C with continuous shaking on a plate reader (Spectramax M5, Molecular Device). The polymerization process was monitored for 110 hours by reading the ThT-fluorescence intensity at 444 nm excitation and 485 nm emission. The time of fibrillization (lag phase) was estimated according to [43], data were analyzed using Origin 8.6 software.

### Cell Culture Experiments

ScGT1 cells were cultivated in 10-cm-diameter plates containing 10 mL of DMEM supplemented with 10% FBS and 1% penicillin-streptomycin, and grown to 95% confluence for 1 week before splitting at 1∶10 for further cultivation. Cells were treated with increasing concentrations of HA or FA (1, 5, 7.5 and 10 µg/mL of HS) and incubated for 6 days at 37°C, 5% CO_2_. PrP^Sc^ accumulation was detected by immunoblotting the PK-digested cell lysates. Five hundred µg of ScGT1 cell lysates total protein was digested by 50 µg/mL of PK (Roche) for 1 hour at 37°C. The reaction was stopped with 5 mM PMSF and the PK-digested cell lysates were ultracentrifuged at 100,000 g for 1 hour at 4°C (Beckman Coulter, Inc.). The pellets were resuspended in 4X loading buffer, boiled for 10 min and immuno-blotted as described above. Fifty µg of ScGT1 was loaded as non-PK control. The half-maximal effective concentration (EC_50_) and the PrP^Sc^ quantification upon HA and FA treatments (at a concentration of 5, 7.5, 10 and 20 µg/mL of HS) were determined using the ELISA assay according to [42]. To evaluate cell viability after HS treatments, we performed the calcein-AM assay (R&D Systems Europe, Ltd.). ScGT1 cells were maintained in DMEM and supplemented with 10% FBS. After one day of incubation, cellular density was determined by cell counting using a haemacytometer. Cell density was adjusted to 2.5×10^5^ cells/mL with DMEM for ScGT1 cells. HA and FA were added to wells at different concentrations (1, 5, 7.5, 10 and 20 µg/mL of HS). Media were aspirated after 6 days of incubation and cells were washed twice with 200 mL of PBS. One hundred µL of 2.5 mM calcein-AM was added and the plates were incubated at 37°C for 30 min. Fluorescence emission intensity was quantified using a fluorescence plate reader (Spectramax M5, Molecular Device) with a 492/525 nm excitation/emission ratio.

## Results

### Insolubility, Folding and Stability of HS-bound Prion Protein

**Figure 1:**
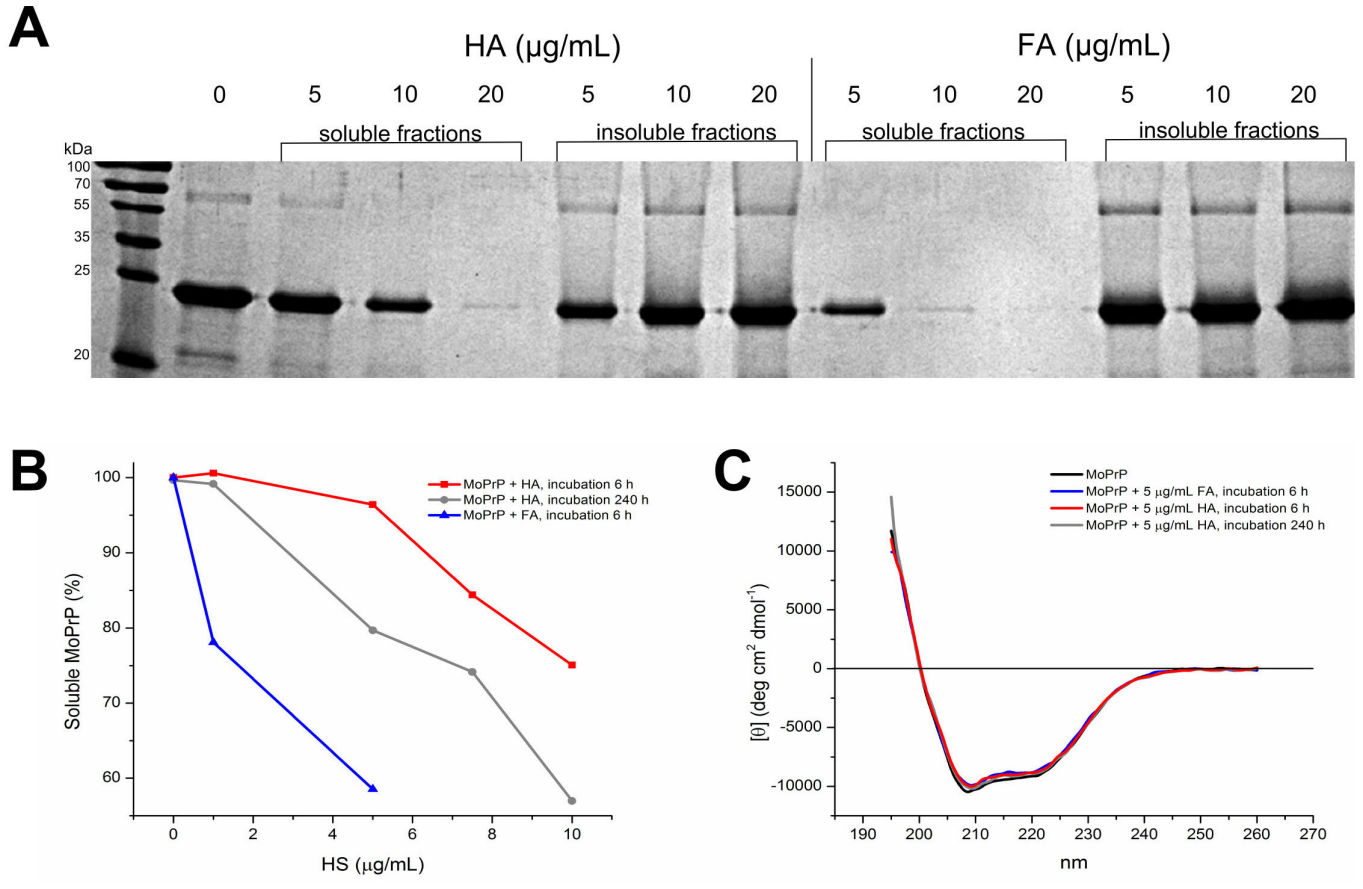
MoPrP adsorption into HS affects protein solubility. SDS-PAGE gel showing the precipitation of full-length MoPrP induced by increasing amounts of HA and FA. After adding 20 and 10 µg/mL of HS (HA and FA, respectively) the total protein is mainly present in the insoluble fractions (A). Decreased MoPrP solubility in the presence of 1 to 5 µg/mL of FA after 6 hours of incubation, and 1 to 10 µg/mL of HA after 6 and 240 hours of incubation (B). Normalized far-UV CD spectra of MoPrP after 6- and 240-hour incubation with 5 µg/mL of HS (C).

To test the interaction between HS and MoPrP, the protein was incubated with increasing concentrations of HA or FA. After 12 hours of incubation with HS, the protein was completely precipitated and present in the insoluble fractions upon addition of up to 20 µg/mL of HS (Figure 1A). Next, we performed CD spectroscopy experiments to evaluate the effect of HS adsorption on MoPrP solubility and folding over time. Differently from other protein detection methods [44], CD spectroscopy detected no interference in HS (Figure S1). Addition of HA or FA causes an increase in millidegree (mdeg) values indicating the gradual MoPrP sequestration from the solution after 6 hours of incubation with HA or FA, and 240 hours of incubation with HA (Figure S1). The correct concentration of soluble MoPrP after HS addition was inferred by a calibration curve (Figure S2). Our results suggest that FA are more effective in inducing MoPrP precipitation, as 97% and 58% of MoPrP was soluble after 6 hours of incubation with 5 µg/mL HA and FA, respectively. After 240 hours of prolonged incubation with HA, the solubility of MoPrP decreased –from 75% of soluble MoPrP incubated with 10 µg/mL HA for 6 hours to 55% incubated for 240 hours. No information was available for long-term FA-MoPrP interaction, because the protein fully precipitates after 6 hours of incubation with 10 µg/mL FA (Figure 1B). Normalized CD spectra revealed that the remaining protein in solution appeared in its native folding, showing the canonical α-helical folded profile of MoPrP with two minima at 208 and 222 nm (Figure 1C) [45].

**Figure 2:**
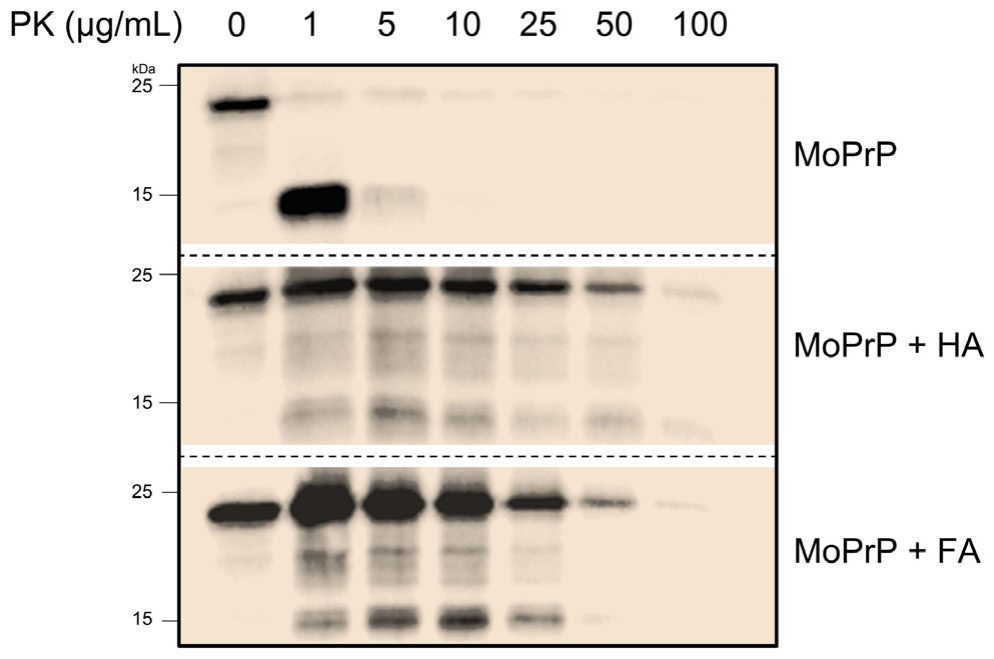
MoPrP adsorbed into HS is protected from PK digestion. Western-blot experiments on MoPrP (first panel), MoPrP-HA and MoPrP-FA complexes (second and third panel, respectively) incubated with different PK concentrations.

To better understand how prions can persist in the environment for several years withstanding biotic or abiotic degradation we tested MoPrP embedded in HS for resistance to PK digestion. To avoid any possible interference of residual HS in solution and PK activity, precipitated MoPrP-HS fractions were resuspended twice in acetate buffer before adding the PK. Unlike the protein alone, HS-adsorbed MoPrP is highly resistant to proteolytic digestion after adding up to 50 µg/mL of PK (Figure 2). At low PK concentrations, FA appear more effective than HA in protecting MoPrP from degradation.

### Optical Microscopy and AFM Characterization of MoPrP-HS Macrocomplexes

**Figure 3:**
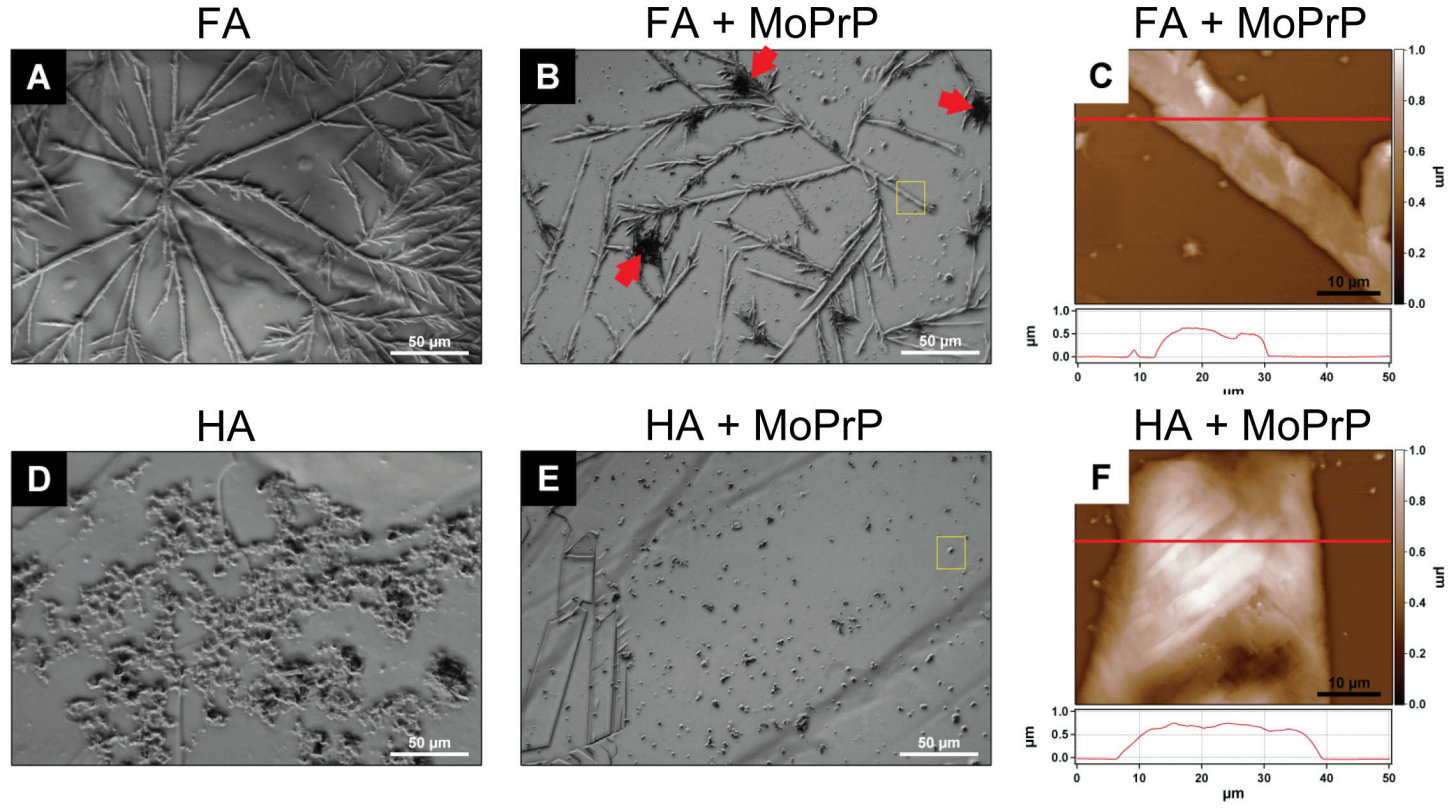
Macroscopic characterization of humic substances. Phase contrast optical images of HS morphology when deposited on a mica surface (20 µg/mL of FA and HA, respectively in A and D) were compared to MoPrP-HS complexes morphology deposited under the same conditions (B shows complexes between 60 µg/mL of MoPrP and FA, E depicts those with HA). Red arrows in B point at “brush-like” structures possibly ascribable to MoPrP clusters. Yellow squares highlight the FA and HA structures analyzed by AFM in panels C and D, representing three-dimensional AFM reconstructions of FA-MoPrP fractal fibers and HA-MoPrP assemblies, respectively.

To further characterize the protein-HS insoluble adducts we analyzed their morphology by optical microscopy and AFM. The macroscopic architectures of FA and HA deposited on mica at 20 µg/mL appeared very different. FA displayed regular, long branched chain-like structures forming a fibrous network fairly distributed on the surface (Figure 3A). Similar regular assemblies were observed in FA-MoPrP complexes, although smaller aggregated particles co-localizing with FA branches were visible (Figure 3B red arrow and Figure 3C). Compared to FA, the optical microscopy analysis on HA revealed a heterogeneous morphology, with large aggregate assemblies (Figure 3D). These morphological differences may be attributed to the different physicochemical nature of FA and HA [27]. The adsorption of MoPrP to HA seems to affect the overall distribution of the adducts on the mica surface (Figure 3E), resulting by AFM in a polydisperse distribution of discrete globular clusters with height and length of 0.6 µm and 30 µm, respectively (Figure 3F).

**Figure 4:**
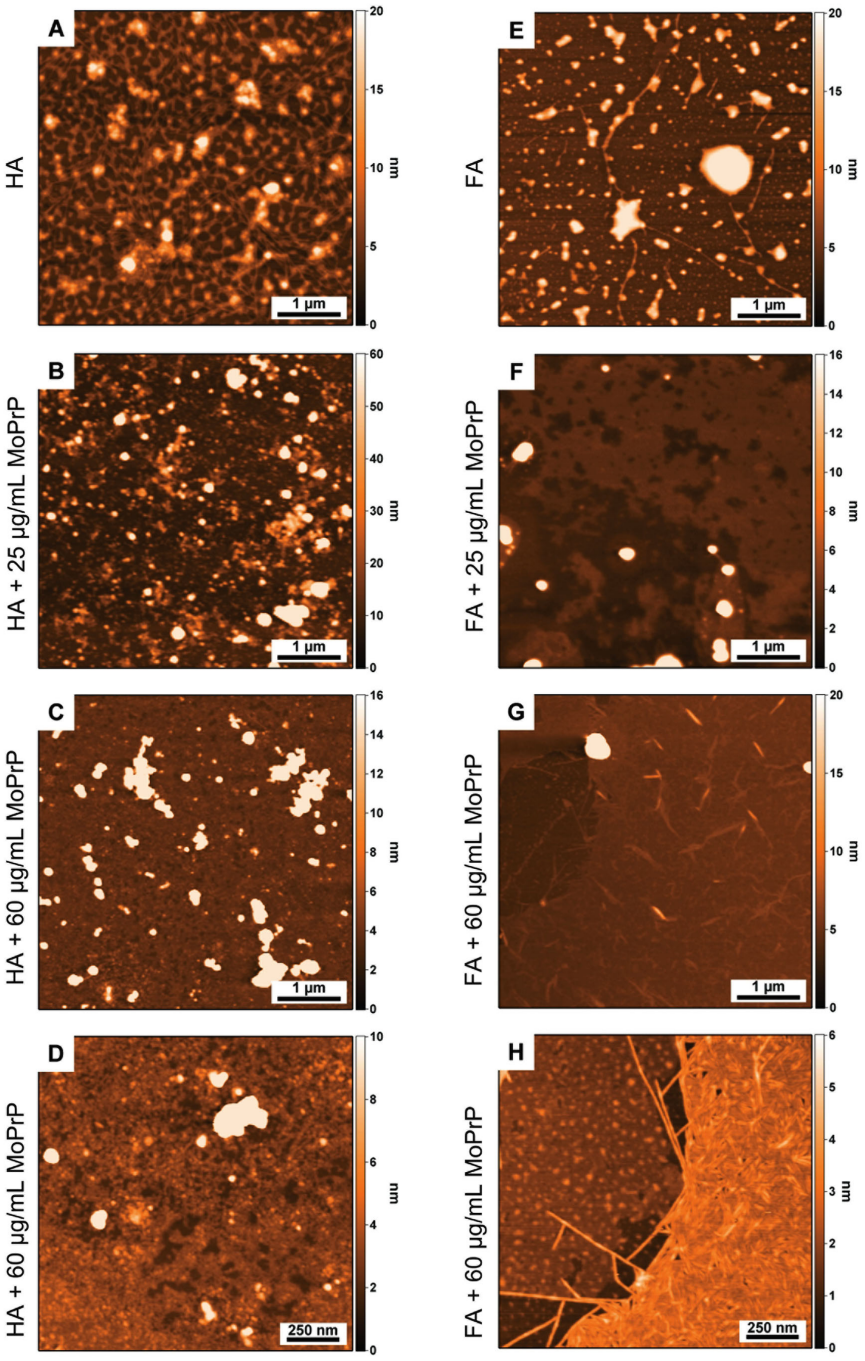
AFM characterization of HS and MoPrP-HS complexes. Surface morphology of HS alone (HA and FA in panels A and E, respectively) and HS complexes with 25 µg/mL of MoPrP (in panels B and F corresponding to MoPrP-HA and MoPrP-FA complexes, respectively) and with 60 µg/mL of MoPrP (in panels C and G with HA and FA, respectively). Panels D and H show high magnification images of the samples presented in panels C and G.

Next, we investigated by AFM the surface regions that appeared devoid of the large FA branched chains or HA aggregates observed by optical microscopy. HA showed aggregated structures connected by gel-like filaments (Figure 4A) while FA samples revealed the presence of globular clusters, which appeared as gel-like clumps (Figure 4E). These morphologies were presumably induced by strong water-HS interaction due to HS surface charges.

HA and FA were further examined in the presence of two different MoPrP concentrations (25 and 60 µg/mL) in order to evaluate possible HS morphological changes induced by the protein. After MoPrP adsorption into HA, the assemblies became more compact and heterogeneous, forming supramolecular clusters in a protein concentration-dependent manner (Figure 4B–D). Interestingly, in the presence of MoPrP, FA associates in a completely different way. At low protein concentration (25 µg/mL) a sponge-like structure on the surface was visible (Figure 3F) suggesting a MoPrP effect on FA assembly. This morphology appeared to rearrange in the complex formed by 60 µg/mL of MoPrP and FA. The porous layer-like structures were significantly narrowed and small ordered assemblies were visible (Figure 4G). A detailed analysis revealed fibrillar structures with different topology, arranged as a thin film with a distribution between 3 and 6 nm in height (Figure 4H). FA fibrils were as long as 1 µm and composed of straight ribbon-like fibrils and fibrils with occasional branching. The globe-shaped layer in panel D is about 1.8±0.2 nm high, while fibers in panel H have a height of about 1.6±0.4 nm. MoPrP deposited as control at 60 µg/mL showed a homogeneous layer about 1.4±0.2 nm high, as determined from a scratch made in the layer (Figure S3A). Next we evaluated the morphological changes of the adducts by decreasing the HS concentration to 5 µg/mL, a condition that does not induce a complete protein precipitation. The AFM scans showed initial rearrangements of the assemblies formed upon addition of HA or FA. The MoPrP-HA complex appeared as an amorphous layer uniformly dispersed on the surface, with an average height of 6 nm (Figure S3B). Conversely, the MoPrP-FA complex formed spherical globular clusters with a distribution of 9 nm in height (Figure S3C), confirming the ability of the FA-MoPrP complex to arrange in regular structures.

### Effectiveness of HS in Inhibiting Prion Replication

**Figure 5:**
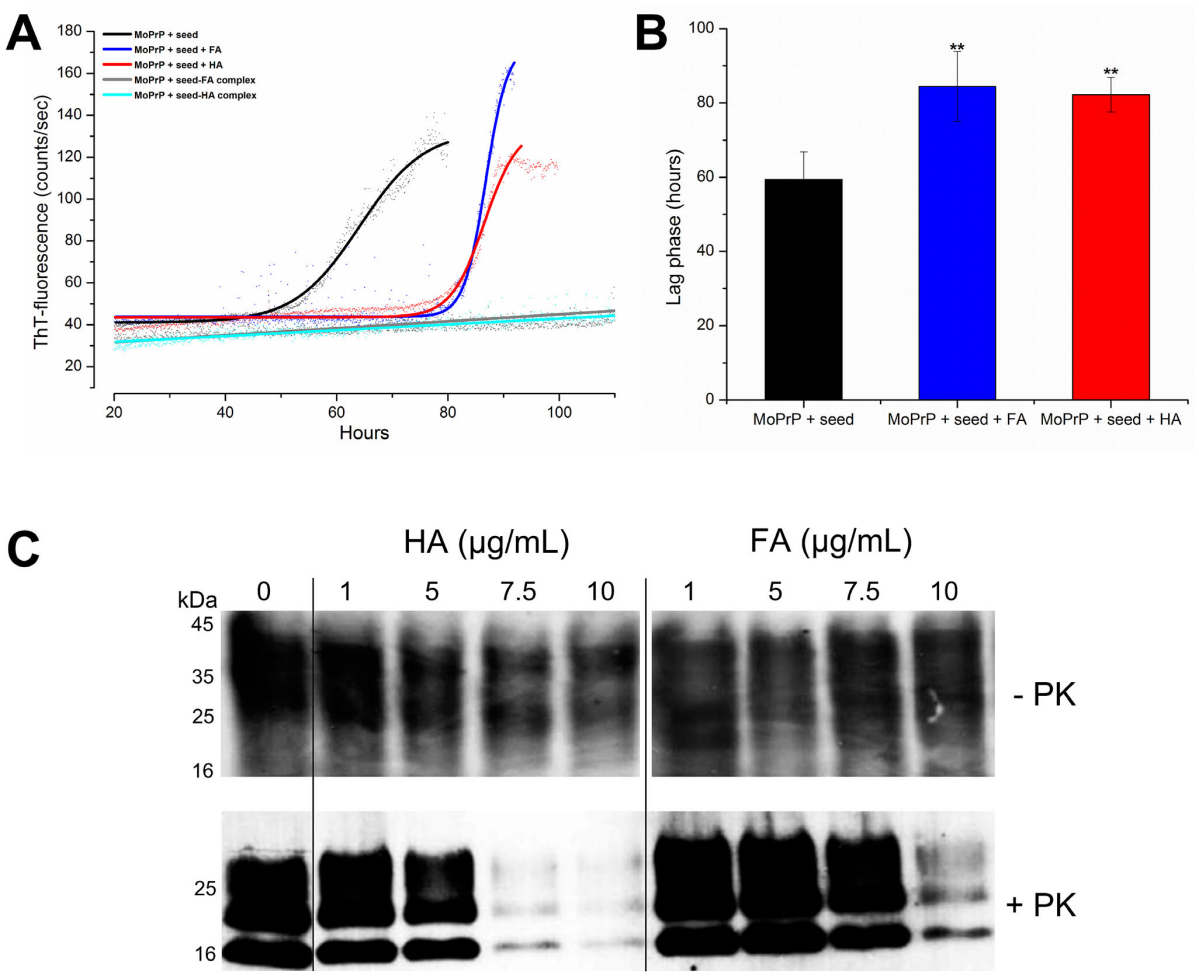
Effectiveness of HS in inhibiting prion replication. ASA showing the kinetics of MoPrP fibrillization in the presence of 1 µg/mL of HS (A) and the corresponding mean value of the lag phases (in hours) for MoPrP treated with HS (**P<0.01) (B). In (C) Western-blot results showing the dose-dependent removal of PrP^Sc^ from ScGT1 cells. In the upper panel, total PrP expression detected by immunoblotting after the addition of increasing concentrations of HA and FA (here used as non-PK controls); the lower panel shows PrP^Sc^ levels after digestion with PK.

To investigate the possible role of HS in reducing prion infectivity, first we used the amyloid seeding assay (ASA). In contrast to the original protocol employing denaturants and high ionic strength to accelerate the fibrillization reaction [37], we used MoPrP in native conditions in the presence of 1 µg/mL of HS. This concentration has a minor impact on protein solubility (Figure 1) and does not interfere with Thioflavin-T (ThT) fluorescence, the dye commonly used in ASA to monitor the formation of newly generated β-strand-enriched structures. When the protein was pre-incubated with PrP^Sc^ and then exposed to HA and FA, the lag-phases of fibrillization were prolonged up to 88.2±4.7 and 84.5±9.4 hours, respectively, while the control started to polymerize after 59.4±7.4 hours. To exclude that the observed lag-phase shifts were due to a partial MoPrP removal from the reaction induced by HS encapsulation, we preincubated the seed with HS before adding MoPrP. Interestingly, in these conditions we did not detect any ThT-fluorescence sigmoidal increases in the reactions seeded by PrP^Sc^ complexed by HS (Figure 5A and 5B). We obtained similar results, showing no ThT-fluorescence increase, in control experiments performed in the presence of HS without PrP^Sc^ seed (Figure S4). To investigate whether HS have a significant effect in reducing PrP^Sc^ content in more physiological conditions, we added HS to ScGT1 cells medium and evaluated PK-resistant PrP^Sc^ levels, a test currently employed to assess whether a compound displays anti-prion activity [38]. As shown in Figure 5C, the treatment with HA and FA induced a clearance of pre-existing PrP^Sc^ from ScGT1 cells in a dose-dependent manner (*lower panel*) without altering the total PrP^C^ expression (*upper panel*). The half maximal effective concentrations (EC_50_) were 7.8±0.4 and 12.3±0.7 µg/mL for HA and FA, respectively, as determined by ELISA [42]. None of the compounds tested was cytotoxic after HS exposure (Figure S5). These results provide first evidence that HS may play a role in PrP^Sc^ adsorption, thus limiting prion transmission.

## Discussion

Similarly to mineral components, organic matter significantly affects soil physico-chemical properties, which may impact PrP^Sc^ adsorption capacity and its stability in the environment.

As experimental model to describe the protein-HS interactions here we used the full-length murine recPrP as surrogate for PrP^Sc^. Although MoPrP is not equivalent to PrP^Sc^, as it has a different structure and lacks glycosylation, it may provide important clues about the protein stability inside HS and the mechanism of protein encapsulation mediated by HA and FA. Our findings show that HS are potent protein complexants that can form insoluble assemblies. The stability of MoPrP adsorbed into HS was confirmed by SDS-PAGE analysis showing no HS-mediated abiotic degradation processes, as also previously reported in recPrP-catechol interaction studies [35]. Because the chemical formulas and molecular weights of HS are still controversial issues [25], the direct comparison between HA and FA was precluded. Therefore, we obtained semi-quantitative data about MoPrP adsorption and folding upon HS interaction. CD experiments on soluble MoPrP exposed to HS revealed that the protein secondary structure remained unaltered. Limited information is available about the folding of the insoluble HS-adsorbed MoPrP. However, it is plausible that encapsulated MoPrP retains its native folding, as observed in the fibrillization experiments where HS were not able to promote MoPrP conversion in β-sheet enriched structures. Moreover, previous experiments using Fourier infrared spectroscopy and cross-polarization magic angle spinning (CP MAS) ^13^C-solid state NMR spectroscopy have reported no detectable conformational changes occurring on recPrP adsorbed in humic-like substances, in spite of large background noise given by carbon groups naturally present in HS [34], [35], [46]. HS also affect the resistance of MoPrP to biotic degradation, as reported in our controlled PK digestion experiments. We argue that prions may be adsorbed by HS in soil rich in OM content and at least partially hidden by natural proteases, thus persisting in the environment for years. This observation is in agreement with current studies proposing that embedding into HS promotes protein preservation in natural environments. On the other hand, this may reduce HS-embedded protein activity and bioavailability [30], [31].

We have provided a morphological description of MoPrP-HS insoluble complexes by optical microscopy and AFM. These techniques have been employed to study the topography and conformational structures of HS on solid surface [47]–[49] as well as protein complexes formed with soil or HS [23], [50]. We used mica as substrate because its surface properties are similar to those of minerals present in terrestrial environments. Differently from HA and HA-MoPrP, FA and FA-MoPrP complexes exhibit ordered structures forming large branches fairly distributed on the surface. The lower molecular weight of FA and their higher oxygen content compared to HA –which are rather a complex mixture of different acids with a variety of functional groups [27]– enable these acids to arrange in regular structures depending on FA concentration and on the presence of proteins or other ligands [48], [51]–[53]. The peculiar structural plasticity of FA was clearly visible in AFM experiments showing sponge-like structures and globular assemblies at low MoPrP and FA concentrations, respectively, while at high MoPrP concentration FA appeared assembled in fibrillar structures. Although AFM provides only morphological information, these fibrils are reasonably made only of FA, as their height is not compatible with current studies on PrP fibrils dimension [43], and as FA cannot generate ThT-positive MoPrP aggregates. Taken together, these findings indicate that protein encapsulation by HS causes the formation of large insoluble aggregates, as is the case with HA, or ordered structures made of FA. Interestingly, FA appear more effective in inducing MoPrP precipitation and in protecting the protein from PK digestion, arguing for a stronger molecular interaction with MoPrP, which results in a different, more efficient encapsulation.

The observed ability of HS to promote MoPrP adsorption raises the question whether they can interact in a similar way also with PrP^Sc^. It has been reported that HS may form adducts with proteins having biochemically different features [29], [31], [54] and a recent study has shown a direct interaction between different types of HA and two PrP^Sc^ strains, isolated from hamster-adapted transmissible mink encephalopathy and from CWD-affected deer [55]. Our findings, based on ASA and ScGT1 cell assays, support the hypothesis that HS effectively hinder prion conversion and infectivity. The observation that HS interact with α-helix folded MoPrP provides a molecular explanation for their effect in inhibiting MoPrP fibrillization and in clearing PrP^Sc^ from ScGT1.

Therefore, we argue that prions may be strongly retained in soils with higher HS content, thus reducing the odds of infectivity among grazing lands. HS-mediated adsorption may promote prion preservation from biotic and abiotic degradation; on the other hand, HS might also limit prion bioavailability to animals exposed to contaminated soils. Previous studies show that prions bound to a soil rich in OM (*e.g.* Dodge soil) are less infectious than PrP^Sc^ adsorbed to montmorillonite [20]. As shown by mild thermal denaturation, FA inhibit the conversion of recPrP to a PK-resistant PrP^Sc^-like conformation, which is not available for cell internalization when bound to FA [32]. A recent study has reported that HA-adsorbed PrP^Sc^ strains resulted slightly less infectious when intracerebrally inoculated in hamsters [55]. Nevertheless, further investigations in living organisms are needed to verify this hypothesis. Our data provide a platform for ongoing, rationally designed experiments aimed at elucidating whether mammalian prions adsorbed into HS are still infectious when orally transmitted in animal models.

## Supporting Information

Figure S1 **Adsorption of MoPrP into HS monitored by CD spectroscopy.** Decrease in millidegree (mdeg) absorbance on the CD spectra of MoPrP after 6 hours of incubation with HA (A), FA (B) and 240 hours of incubation with HA (C).
(TIF)

Figure S2 **Estimated concentration of MoPrP in solution after HS addition using MoPrP standards.** CD spectra of MoPrP standards (125, 100, 75 and 50 µg/mL) were acquired (A) and the equation resulting from the linear fitting of the mdeg value at 208 nm *vs.* the MoPrP standards was derived (B).
(TIF)

Figure S3 **AFM surface morphology of MoPrP in complex with low HS concentration.** AFM surface reconstruction of a MoPrP solution (60 µg/mL) drop-casted on a freshly cleaved mica surface (A). In B and C, respectively, surface characterization of the MoPrP-HA and MoPrP-FA complexes formed by 60 µg/mL of MoPrP and 5 µg/mL of HA or FA. Height profiles, marked by red lines on panels, evidenced a flat layer in A characterized by a height of about 1.4±0.2 nm, whereas a more globular morphology appeared in panels B and C.
(TIF)

Figure S4 **Fibrillization reactions of MoPrP in the presence of 1 µg/mL HS without PrP^Sc^ addition.** No significant sigmoidal ThT-fluorescence increases were detectable in the tested conditions.
(TIF)

Figure S5 **Viability experiments on ScGT1 cells treated with FA (upper panel) and HA (lower panel) as evaluated in the calcein-AM assay.**
(TIF)
