# Supplementary figures and images for: Prion Protein Interaction with Soil Humic Substances: Environmental Implications
Source: PLoS One. 2014 Jun 17;9(6):e100016. doi: 10.1371/journal.pone.0100016 (PMC4061048; doi:10.1371/journal.pone.0100016)

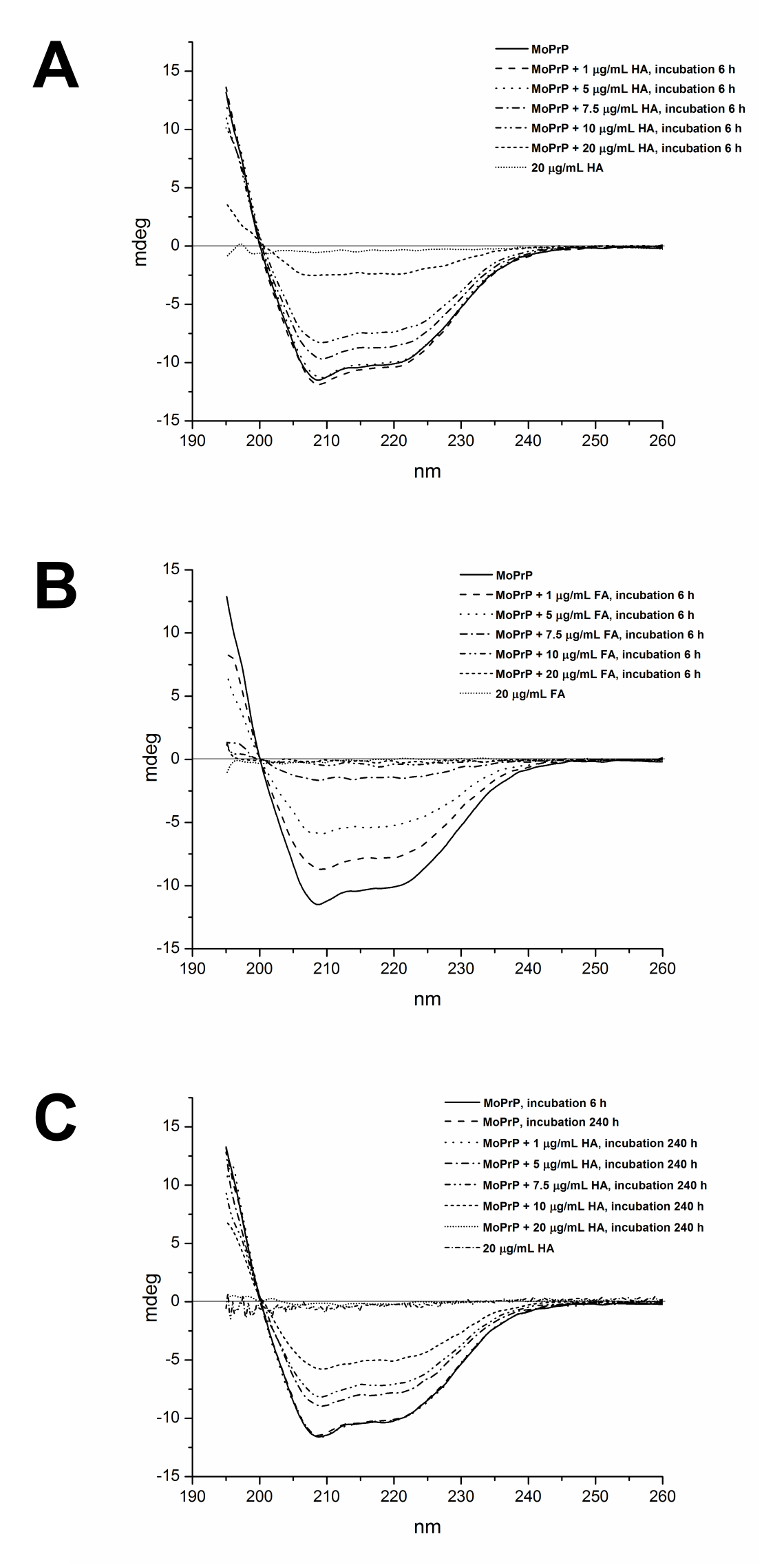

Supplement: Figure S1 — Adsorption of MoPrP into HS monitored by CD spectroscopy. Decrease in millidegree (mdeg) absorbance on the CD spectra of MoPrP after 6 hours of incubation with HA (A), FA (B) and 240 hours of incubation with HA (C). (TIF) [file pone.0100016.s001.tif]

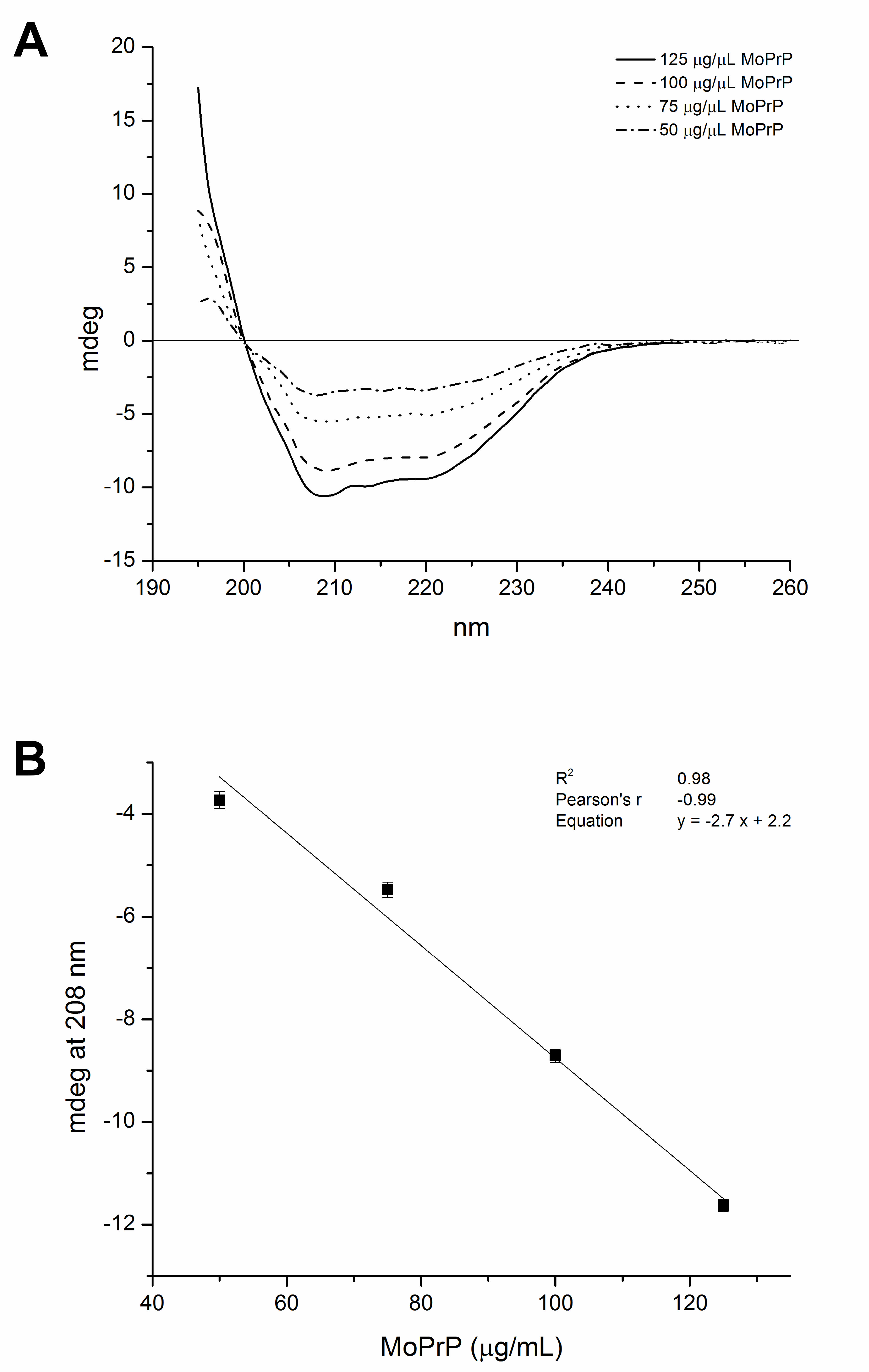

Supplement: Figure S2 — Estimated concentration of MoPrP in solution after HS addition using MoPrP standards. CD spectra of MoPrP standards (125, 100, 75 and 50 µg/mL) were acquired (A) and the equation resulting from the linear fitting of the mdeg value at 208 nm vs. the MoPrP standards was derived (B). (TIF) [file pone.0100016.s002.tif]

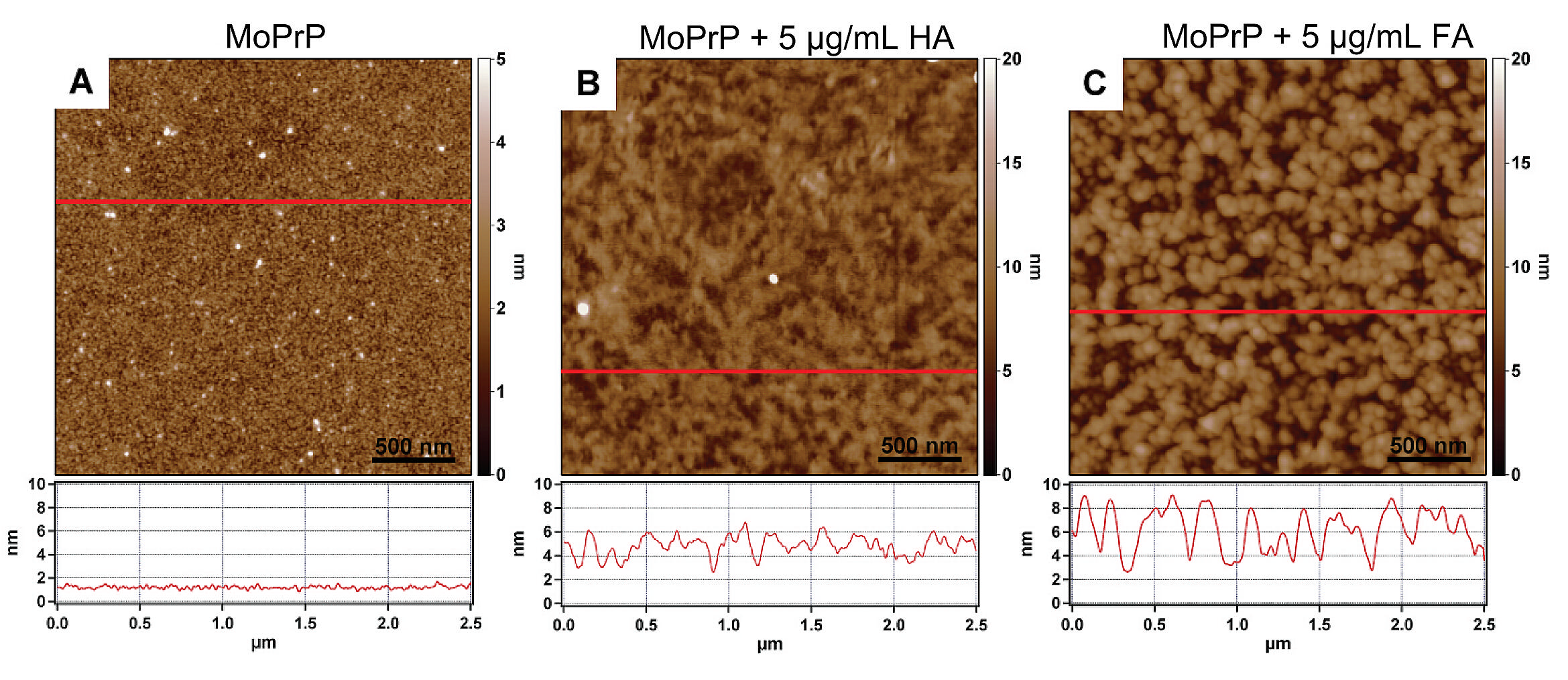

Supplement: Figure S3 — AFM surface morphology of MoPrP in complex with low HS concentration. AFM surface reconstruction of a MoPrP solution (60 µg/mL) drop-casted on a freshly cleaved mica surface (A). In B and C, respectively, surface characterization of the MoPrP-HA and MoPrP-FA complexes formed by 60 µg/mL of MoPrP and 5 µg/mL of HA or FA. Height profiles, marked by red lines on panels, evidenced a flat layer in A characterized by a height of about 1.4±0.2 nm, whereas a more globular morphology appeared in panels B and C. (TIF) [file pone.0100016.s003.tif]
